# Regulation of developmental and environmental signaling by interaction between microtubules and membranes in plant cells

**DOI:** 10.1007/s13238-015-0233-6

**Published:** 2015-12-19

**Authors:** Qun Zhang, Wenhua Zhang

**Affiliations:** College of Life Sciences, State Key Laboratory of Crop Genetics and Germplasm Enhancement, Nanjing Agricultural University, Nanjing, 210095 China

**Keywords:** abiotic stresses, cortical microtubule, lipids, plasma membrane

## Abstract

Cell division and expansion require the ordered arrangement of microtubules, which are subject to spatial and temporal modifications by developmental and environmental factors. Understanding how signals translate to changes in cortical microtubule organization is of fundamental importance. A defining feature of the cortical microtubule array is its association with the plasma membrane; modules of the plasma membrane are thought to play important roles in the mediation of microtubule organization. In this review, we highlight advances in research on the regulation of cortical microtubule organization by membrane-associated and membrane-tethered proteins and lipids in response to phytohormones and stress. The transmembrane kinase receptor Rho-like guanosine triphosphatase, phospholipase D, phosphatidic acid, and phosphoinositides are discussed with a focus on their roles in microtubule organization.

## INTRODUCTION

In plants, the cytoskeleton consists of two main components: microtubules and actin filaments. Specific cytoskeleton configurations are required for diverse essential processes such as chromosome segregation, intracellular transport, cell motility, and cell shape determination (Hashimoto, 2015). Organization of the interphase cortical microtubule array, which is anchored tightly to the plasma membrane, guides plant growth and morphogenesis by acting in cell division and polarity, and in responses to abiotic stresses (Lindeboom et al., 2013; Pleskot et al., 2013, 2014). The dynamic nature of microtubules provides the flexibility to rearrange them into different arrays in response to developmental and environmental stimuli (Wang et al., 2007, 2012; Zhang et al., 2012). To support these diverse functions, the cortical microtubule arrays are accurately organized by microtubule-associated proteins and lipids in the plasma membrane (Zhang et al., 2012; Pleskot et al., 2013).

Understanding how cortical microtubules are organized into specific array patterns and the underlying molecular mechanisms remains a challenge (Lucas and Shaw, 2008; Hamada, 2014). Real-time observations of microtubule dynamics in axially growing cells, in combination with analysis of phospholipid regulation of cytoskeletal organization, have provided a deep appreciation of the regulatory networks involved in cytoskeletal organization (Lin et al., 2014; Pleskot et al., 2014). Cytoskeletal dynamics and its regulation have been the subject of multiple reviews (Dixit and Cyr, 2004; Lloyd and Chan, 2004; Ehrhardt and Shaw, 2006; Pleskot et al., 2013, 2014). In this review, we describe recent advances in elucidating the functions of cortical microtubules in response to phytohormones and abiotic stresses, and their functional regulation by membrane-associated and membrane-tethered proteins and lipids.

## MICROTUBULE FUNCTIONS IN HORMONE-MEDIATED DEVELOPMENTAL PROCESSES

### Microtubule reorganization and auxin response

**Figure 1:**
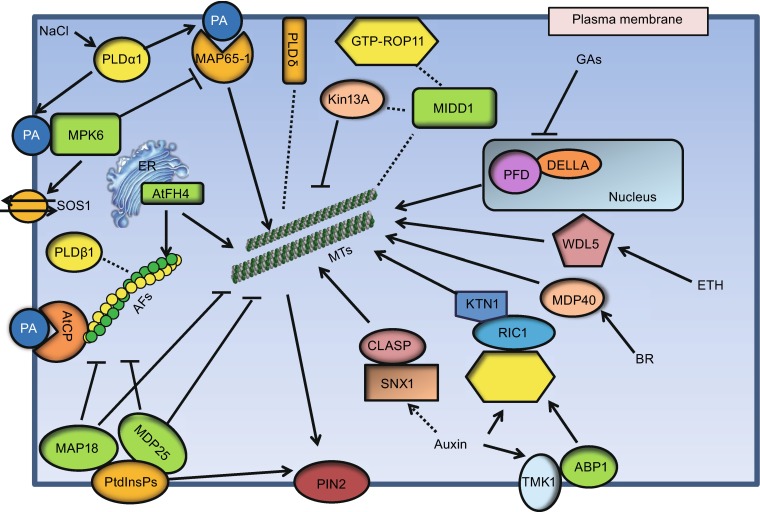
**Model of cortical microtubule organization regulated by membrane-associated proteins and lipids in response to plant hormones and stress**. Activation of PLDα1 by salt stress leads to production of PA, which binds to MAP65-1 and MPK6 to regulate microtubule organization and the SOS pathway, respectively. PA also binds to AtCP to modulate actin filaments. The PtdInsPs-binding proteins, MAP18 and MDP25, negatively regulate cytoskeletal organization. Plasma membrane-localized TMK1 interacts with ABP1 and transduces auxin signals to activate plasma membrane-associated ROPs leading to changes in the cytoskeleton and the shape of leaf pavement cells. Locally activated ROP11 recruits MIDD1 to plasma membrane domains. MIDD1 links plasma membrane, cortical microtubules, and Kin13A. CLASP regulates levels of the auxin efflux carrier PIN2 by tethering SNX1 endosomes to cortical microtubules, which fine tunes auxin maxima in the root apical meristem. In the absence of GA, DELLA retains PFD in the nucleus, and in the presence of GA, DELLA proteins are degraded, which allows PFD to move to the cytoplasm leading to increased production of tubulin dimers. WDL5 and MDP40 act as important mediators in regulating microtubule organization in response to ETH and BR signals, respectively. ER, endoplasmic reticulum; MTs, microtubules; AFs, actin filaments

Auxin participates in various developmental processes. One major effect of auxin is cell expansion, which relies on the coordinated activities of cellular processes involving microtubules (Ruan and Wasteneys, 2014; Adamowski and Friml, 2015). When cells elongate, cortical microtubules are arranged perpendicularly to the axis of cell elongation (transverse microtubules), while a longitudinal alignment induces growth inhibition. In response to auxin, root microtubules change from transverse to longitudinal, inhibiting cell expansion (Chen et al., 2014). Using the TILLING mutant, which is defective in AUXIN BINDING PROTEIN1 (ABP1) (*abp1-5*), it was further demonstrated that the effect of auxin requires ABP1 and involves the contribution of downstream signaling components, including Rho-like GTPase from plants 6 (ROP6), and the ROP-interacting protein RIC1 (Lin et al., 2013; Chen et al., 2014). In leaf pavement cells of *Arabidopsis*, the plasma membrane-localized transmembrane kinases (TMKs) belonging to the receptor-like kinase family has been found to interact with ABP1. The TMK-ABP1 interaction is required to activate ROPs, which play a role in regulating cytoskeleton organization and the endocytosis of PIN-FORMEDs (PINs), which are auxin efflux carrier proteins (Xu et al., 2014; Fig. 1).

Overall, the stated functions of ABP1 are inconsistent. A viable *abp1-5* TILLING allele was used to identify the functions of ABP1, including the auxin-responsive rearrangement of microtubules, PIN protein internalization, and other molecular and cellular processes (Robert et al., 2010; Baster et al., 2013; Effendi et al., 2013; Chen et al., 2014; Paque et al., 2014; Xu et al., 2014). More recently, however, Gao et al. (2015) used ribozyme-based CRISPR technology to generate an *abp1* mutant with a 5-bp deletion in the first exon of ABP1, and they isolated a T-DNA insertion *abp1* allele. None of the mutants showed either auxin signaling or developmental phenotypes. Furthermore, genome sequencing of the *abp1-5* mutant revealed that background mutations may lead to auxin and other phenotypes (Enders et al., 2015). Complementation tests and a re-valuation of the functions of ABP1 have been proposed for the future work; additional information about ABP1 can be found in other reports (Enders et al., 2015; Liu, 2015 ).

Cortical microtubules in turn influence polar auxin transport (Heisler et al., 2010; Ambrose et al., 2013; Zhang et al., 2013; Ruan and Wasteneys, 2014). Short-term treatment with the microtubule-disrupting drug oryzalin had no effect on the polarity of PIN proteins (Boutte et al., 2006; Geldner et al., 2001); however, prolonged oryzalin treatment interfered with basal PIN2 targeting in young cortical cells and with PIN1 targeting in the stele, resulting in reduced polar distribution (Kleine-Vehn et al., 2008). The *Arabidopsis* microtubule-associated protein CLASP interacts with the retromer component sorting nexin 1 (SNX1) protein to mediate the association between endosomes and microtubules. Plants carrying the *clasp-1* mutation display enhanced PIN2 degradation and aberrant auxin distribution, which is promoted by microtubule depolymerization (Ambrose et al., 2013; Brandizzi and Wasteneys, 2013). These findings indicate that intact microtubules are required for the polar distribution of PIN proteins and auxin function.

### Microtubules, stomatal development, and abscisic acid signaling

Stomatal morphogenesis takes place after the symmetric division of a guard mother cell, followed by the development of wall thickening in each daughter cell and their separation to form the stomatal pore in a microtubule-dependent process (Galatis and Apostolakos, 2004; Lucas et al., 2006). The highly organized microtubules in *Arabidopsis* stomatal cells play key roles in the morphogenesis of stomatal complexes (Galatis and Apostolakos, 2004; Lucas et al., 2006). The preprophase bands (PPBs) of microtubules in mature mother cells are located away from stomata, and radially oriented microtubules converge near the central rim of the stomatal pore, suggesting an essential function of microtubules in asymmetric division (Lucas et al., 2006). Mutations in *Arabidopsis* MUSTACHES (MUS), a leucine-rich repeat receptor-like kinase, disrupt stomatal symmetry resulting in stomatal defects and depolarized radial microtubule arrays (Keerthisinghe et al., 2015).

Reorganization of the cortical microtubule cytoskeleton is critical for guard cell function, particularly in the abscisic acid (ABA) signaling pathway (Marcus et al., 2001; Eisinger et al., 2012a, b; Jiang et al., 2014). An apparent loss of microtubules was observed in guard cells upon stomatal closure, probably due to microtubule instability or rearrangement. The depolymerization of guard cell microtubules by oryzalin prevented *Arabidopsis* stomatal opening, while the stabilization of microtubules delayed stomatal closure (Eisinger et al., 2012a). Microtubules were further observed using green fluorescent protein fused to α-tubulin 6 (GFP-TUA6). The total amount of polymerized tubulin was higher in open than in closed guard cells; this was correlated with an increase in the total fluorescence (Eisinger et al., 2012b). These results are in agreement with genetic evidence showing that the mutation of *CONSTITUTIVELY PHOTOMORPHOGENIC 1* (*COP1*), which encodes an *Arabidopsis* RING finger-type ubiquitin E3 ligase, results in tubulin degradation and stomatal closure (Khanna et al., 2014). COP1 has been studied extensively as a critical destabilizer of photomorphogenesis-promoting factors. Because light is an important factor in the regulation of stomatal movement, the finding of a COP1-mediated microtubule array opens a new avenue for understanding the regulatory mechanisms underlying microtubule organization (Mao et al., 2005). Taken together, these results suggest that the microtubule array organization is correlated with and required for stomatal opening and closure. Microtubules may control the activity of plasma membrane ion channels such as those that transport calcium, and lipid signaling may be involved in this process. Phospholipase D (PLD) catalyzes phospholipid hydrolysis to produce phosphatidic acid (PA) and a free head group. It was demonstrated that PLD and PA are involved in the ABA-induced stomatal closure (Zhang et al., 2009). Treatment with calcium induces depolymerization of microtubules and stomatal closure in wild-type *Arabidopsis*, but not in the *pldα1* mutant (Jiang et al., 2014). In addition, both ABA-induced microtubule depolymerization and stomatal closure were impaired in *pldα1*, and cotreatment with ABA and microtubule-disrupting drugs rescued the *pldα1* phenotype (Jiang et al., 2014).

The *cop1* mutation not only induces tubulin degradation, it also impairs the calcium ion-dependent activation of S-type anion channel currents in guard cells, which are activated to drive stomatal closure. However, the *cop1* mutation did not change the activation of inward K^+^ channel currents required for stomatal opening (Khanna et al., 2014). It is still an open question whether S-type anion channels and microtubules may function independently, or whether they act together to regulate stomatal movement.

### Roles of the hormones GA, ethylene, and brassinosteroid in microtubule organization

DELLA nuclear proteins restrain cell proliferation and expansion, leading to inhibited plant growth (Peng et al., 1999), and they integrate salt-activated ethylene (ETH) and ABA signaling in response to environmental changes (Achard et al., 2006). A recent study established DELLA proteins as a mechanistic link between GA and cortical microtubule organization (Locascio et al., 2013). DELLA proteins interact with the prefoldin (PFD) complex, a cochaperone required for tubulin folding (Locascio et al., 2013). In the presence of GA, DELLAs are degraded and the FPD complex is shuttled into the cytoplasm where it produces active tubulin subunits. In the absence of GA, PFD is localized to the nucleus, where it compromises α/β-tubulin heterodimer availability, affecting microtubule organization (Locascio et al., 2013). A loss of function of PFD impairs microtubule organization, rendering the *pfd* mutant hypersensitive to salt stress (Rodriguez-Milla and Salinas, 2009). These results demonstrate that GA-mediated microtubule organization plays an essential role in salt tolerance.

As a gaseous plant hormone, ETH is essential for plant growth and development, including seed germination, leaf senescence, fruit ripening, and responses to environmental stresses (Kendrick and Chang, 2008; Muller and Munne-Bosch, 2015). ETH affects the organization of cortical microtubules in plant cells (Takahashi et al., 2003; Polko et al., 2012). The microtubule-associated protein WAVE-DAMPENED2-LIKE5 (WDL5) is a microtubule-stabilizing protein in *Arabidopsis* (Sun et al., 2015). Treatment with 1-aminocyclopropane-1-carboxylic acid (ACC) significantly enhanced the *WDL5* expression and cortical microtubule stability, resulting in decreased etiolated hypocotyl cell elongation, and the reorganization of cortical microtubules in the *wdl5-1* mutant showed reduced sensitivity to ACC treatment (Sun et al., 2015). The above results suggest that cell elongation depends on the microtubule reorganization, and that stabilized microtubules are required for EHT-inhibited etiolated hypocotyl cell elongation, which involves WDL5 as a positive participant. In addition, WDL3 overexpression resulted in overall shortening of hypocotyl cells and stabilization of cortical microtubules in the light, and WDL3 protein was abundant in the light, but was degraded through the 26S proteasome pathway in the dark (Liu et al., 2013).

Brassinosteroid (BR) mediates hypocotyl cell elongation by a mechanism that may control the orientation and stability of cortical microtubules. The key transcription factor BRASSINAZOLE-RESISTANT1 (BZR1) targets and upregulates microtubule destabilizing protein 40 (MDP40) directly, thereby serving as a positive regulator of hypocotyl cell elongation (Li, 2010; Gudesblat and Russinova, 2011; Wang et al., 2012). Genetic evidence shows that the light/GA-signaling pathway affects the properties of microtubules required to reorient growth (Sambade et al., 2012). *Arabidopsis* AUGMIN subunit 8 (AUG8) is a novel microtubule plus end-binding protein that contributes to light-induced microtubule reorientation and modulates cell elongation (Cao et al., 2013). The studies above suggest the existence of a molecular mechanism of putative crosstalk between phytohormones, microtubule dynamics, and cell elongation in response to light or dark environments.

## MICROTUBULE REORGANIZATION IN RESPONSE TO STRESS

Cortical microtubules are not only targets of signaling, but also actively participate in signal transduction itself. Salt stress induces the rapid depolymerization of microtubules and the formation of a new microtubule network via repolymerization (Wang et al., 2007, 2011; Zhang et al., 2012). SPR1, a microtubule-stabilizing protein, is degraded by the 26S proteasome in response to salt stress, and this degradation is essential for salt stress tolerance (Nakajima et al., 2004; Sedbrook et al., 2004; Wang et al., 2011). Moreover, the *spr1* mutant displays a right-handed helical growth phenotype, and interestingly, mutations of the plasma membrane Na^+^/H^+^ antiporter, SOS1, suppress the helical growth phenotype (Shoji et al., 2006). The root microtubules arrays of *sos1* plants are oriented much more randomly than those of wild-type cells under mild salt treatment (Shoji et al., 2006). A recent study showed that the *Arabidopsis* salt overly sensitive 3 (SOS3) protein plays an important role in salt tolerance through regulation of actin filaments (Ye et al., 2013). These findings indicate that the cytoskeleton interacts with the SOS pathway to signal salt stress in plant cells.

PLDα1-derived PA binds to microtubule-associated protein 65-1 (MAP65-1) and regulates cortical microtubule organization in *Arabidopsis* in response to salt stress (Zhang et al., 2012). Under salt stress, knockout of the *PLDa1* gene causes increased NaCl-induced disorganization of microtubules, which cannot be recovered during or after removal of the stress but can be alleviated by exogenous PA. Further evidence reveals that PA binds to residues 53KRK55, 61KSR63, and 428SK429 of MAP65-1, and that this binding is involved in MAP65-1 binding to microtubules (Zhang et al., 2012). Interestingly, PA also binds to mitogen-activated protein kinase 6 (MPK6) and increases its phosphatase activity, which phosphorylates SOS1 and enhances plant salt tolerance (Yu et al., 2010). In addition, MAP65-1 is phosphorylated by mitogen-activated protein kinase 4 (MAPK4 or MPK4) and MPK6 (Smertenko et al., 2006; Beck et al., 2011), their putative orthologs MAPK NRK1/NTF6 (Sasabe et al., 2006), and cyclin dependent protein kinase (CDPK) (Mollinari et al., 2002). These findings have established the existence of crosstalk among phospholipids, microtubules, and phosphatase in response to a stressful environment (Fig. 1).

## MEMBRANE-MICROTUBULE INTERACTION

### Interplay between cortical microtubules and plasma membrane domains

To ensure the proper spatial and temporal regulation of microtubule dynamics, the activity and binding properties of MAPs are further modulated by upstream signaling molecules. Physical linkages between microtubules and the membrane were recently observed using high-resolution scanning electron microscopy (Barton et al., 2008). Only a few candidate MAPs have been proposed as potential linkers between the plasma membrane and microtubules (e.g., PLD [Gardiner et al., 2001]) and *Arabidopsis* membrane-integrated formin (AtFH4 [Deeks et al., 2010]). A 90 kDa tubulin-binding protein from tobacco was identified as a putative PLDδ based on an activity assay and sequence alignment (Gardiner et al., 2001), and the activation of PLD induced cortical microtubules to release from the plasma membrane and partially depolymerize (Dhonukshe et al., 2003). However, the detailed mechanical functions of PLD on microtubule organization remain to be elucidated. AtFH4 coaligns the endoplasmic reticulum with microtubules and also nucleates filamentous (F)-actin. Although an AtFH4-GFP fusion protein was shown to accumulate at the endoplasmic reticulum (ER), it may be trafficked to the plasma membrane to act as a scaffold for cytoskeletal organization (Deeks et al., 2010).

ROP11 is distributed broadly at the plasma membrane. ROP11, after being activated by Rho of the plant guanine nucleotide exchange factor 4 (ROPGEF4), recruits the microtubule depletion domain 1 (MIDD1) protein to induce the local disassembly of cortical microtubules. Conversely, cortical microtubules eliminate active ROP11 from the plasma membrane through MIDD1 (Oda and Fukuda, 2012). The mutually inhibitory interaction between active ROP domains and cortical microtubules is essential to establish the secondary wall pattern in xylem cells (Oda and Fukuda, 2012, 2013).

### Lipid signaling in plant cells

Phospholipids play a key role in maintaining the bilayer structure of membranes and in separating the cytosol from organelles and the extracellular space. The proportions of phospholipids such as PA, inositol 1,4,5-trisphosphate (InsP_3_), and diacylglycerol (DAG) change rapidly, and together with phospholipid-metabolizing proteins, are involved in plant growth and development (Wang et al., 2014). As a rough approximation, PA, an abundant negatively charged phospholipid, constitutes 1%–4% of total cellular lipids (Stace and Ktistakis, 2006). Although PA does not bind to tubulins *in vitro* (Zhang et al., 2012), it may mediate cytoskeletal organization and dynamics by binding to and modulating cytoskeleton-associated proteins (Pleskot et al., 2013).

We demonstrated that PA acts as a linker between the plasma membrane and microtubules via MAP65-1, which is essential for salt-stress signaling in *Arabidopsis* (Zhang et al., 2012). Under salt stress, *Arabidopsis* PLDα1 is activated to produce PA, which binds to MAP65-1, leading to enhanced microtubule polymerization and bundling activity (Zhang et al., 2012). Exogenous application of PA rescues the salt-sensitive phenotype of microtubules in *pldα1*, but not in *map65-1*, clearly indicating that the PA-MAP65-1 interaction is essential for cortical microtubule organization in response to salt stress (Zhang et al., 2012). The two microtubule-destabilizing proteins, MAP18 (AtPCaP2) and MDP25 (AtPCaP1), bind PtdIns(3,4,5)P_3_ and PtdIns(3,5)P_2_*in vitro* indicating that both proteins are involved in intracellular signaling by regulating microtubule organization and interacting with PtdInsPs (Nagasaki et al., 2008; Kato et al., 2010), although no direct evidence for the involvement of PtdInsPs in the regulation of MAP18 and MDP25 has been reported to date.

Like microtubules, the organization and dynamics of actin filaments are mediated by membrane phospholipids. *Arabidopsis* heterodimeric capping protein (AtCP) binds to the barbed ends of actin filaments (Huang et al., 2003), and this activity is regulated by PA (Huang et al., 2006). The interaction between PA and AtCP renders filament ends more dynamic, which significantly enhances filament-filament annealing and filament elongation from free ends (Li et al., 2012). In a separate report, actin and β-tubulin were pulled down with GFP-PLDδ from *Arabidopsis* suspension cells, suggesting that PLDδ connects microtubules with actin filaments in plant cells (Ho et al., 2009). In tobacco (*Nicotiana tabacum*) pollen, actin interacts with NtPLDβ1, and F-actin enhances, while G-actin inhibits, PLDβ1 activity (Pleskot et al., 2010). Thus, PA regulates microtubules and actin through PA-binding proteins, and PLD directly links microtubules and actin. These results suggest that individual PLD isoforms and their product, PA, anchor the cytoskeleton to specific sites on membranes to reorganize them in response to diverse signals. On the other hand, microtubule depolymerization induced by oryzalin activates PLDα1, and depolymerized G-actin inhibits PLDβ1 activity, indicating feedback regulation of PLD activity (Pleskot et al., 2010; Zhang et al., 2012).

## CONCLUSIONS AND OUTLOOK

Microtubules in plant cells are regulated by multiple MAPs to enrich the scope of microtubule behavior, and some MAPs are bound tightly to the plasma membrane (Gardiner et al., 2001; Ambrose and Wasteneys, 2008; Gu et al., 2008; Li et al., 2011). Cortical microtubules and the plasma membrane reorganize themselves and transduce external stimuli to internal systems. Most of these interactions are mediated by membrane-based molecules and microtubule linker proteins. The acidic phospholipid PA, a minor, dynamic component of the bilayer, does not bind to tubulins *in vitro*; instead, it may mediate microtubule organization by interacting with MAPs (Zhang et al., 2012). Moreover, other acidic phospholipids such as phosphatidylserine (PS), phosphatidylglycerol (PG), and phosphatidylinositol (PI) may also mediate cytoskeletal organization directly or regulate MAPs activity involved in microtubule arrays, but supporting evidence remains limited (Pleskot et al., 2013). Therefore, further study is needed to identify additional MAPs that bind to the plasma membrane and phospholipids and interact with cytoskeleton-associated proteins in plant cells.
